# Platelet-rich plasma as an adjuvant in the endometrial preparation of patients with refractory endometrium

**DOI:** 10.5935/1518-0557.20180009

**Published:** 2018

**Authors:** Aixa Molina, Jose Sánchez, William Sánchez, Vanessa Vielma

**Affiliations:** 1 Unidad de Asistencia Materno Reproductiva (UNAMATER) Mérida. Venezuela.; 2 Instituto Venezolano de Fertilidad Caracas (IVF CARACAS) Caracas D.C. Venezuela.

**Keywords:** Platelet-rich plasma, refractory endometrium, endometrial thickness

## Abstract

**Objective:**

To improve endometrial quality and implantation rates after the administration of platelet-rich plasma in patients with refractory endometrium.

**Methods:**

19 patients undergoing *in vitro* fertilization, aged between 33 and 45 years with a history of refractory endometrium, to whom platelet rich plasma was given by infusion with a catheter into the uterine cavity on the tenth day of the hormone replacement therapy, and then 72 hours after the first administration.

**Results:**

Endometrial thicknesses >7mm was reported with the first use; and in all cases, endometrial thicknesses >9mm were evident after the second administration. The entire study group qualified for Embryo Transfer at the blastocyst stage. We had 73.7% of positive pregnancy tests, of which 26.3% yielded live births; 26.3% ongoing pregnancies; 10.5% biochemical pregnancies; 5.3% anembryonic pregnancies and 5.3% had fetal death (16 weeks).

**Conclusions:**

Platelet-rich plasma and its biostimulation effects on the endometrial microvasculature seems to be beneficial to patients with refractory endometrium, providing an increase in endometrial receptivity and a consequent increase in implantation rates. As an autologous resource, they are easy to obtain and inexpensive. Thus, we recommend it to be included in the different protocols for endometrial preparation, including those in which a natural cycle is preferred.

## INTRODUCTION

The refractory endometrium, characterized by atrophy with endometrial interface measurements below 6 mm by ultrasound; and/or the presence of intrauterine adhesions and synechia through hysteroscopy, which is the so-called Asherman syndrome, continues to represent a serious problem for the practitioner who does the endometrial preparation for patients undergoing Assisted Reproductive Techniques (ART). This type of endometrial pattern is still responsible for many embryo transfer (ET) cancellations or implantation failures (Coughlan *et al*., 2014; Kasius *et al*., 2014). Refractory endometrium can be idiopathic, congenital, surgical (uterine curettage); caused by inflammatory processes, infections and radiation therapy, among other causes (Revel, 2012). These different etiological agents cause an important lesion on the basal layer of the endometrium, with consequent decrease in its regeneration capacity. Hormone replacement therapy (HRT) with estradiol valerate, has been registered in the endometrial preparation of infertile patients, requiring low and high complexity treatments. However, other drugs have been added to it such as: acetylsalicylic acid (aspirin); Sildenafil, vitamin E, GnRH agonists, HCG, l-arginine, pentoxifylline, and others, to ensure an adequate implantation window and to promote the synchronization between a prepared embryo to implant and a receptive endometrium (Qublan *et al*., 2008; Takasaki *et al*., 2010; Coughlan *et* *al.*; 2014). Recently, growth factors, mainly G-CSF, stem cells, PRP and bone marrow have been used as alternative treatment modalities; and the extreme resource of uterine transplantation has been tested (Ohl *et al*., 2002; Del Priore *et al*., 2013; Barad *et al*., 2014; Kunicki *et al*., 2014; Singh *et al.*, 2014; Chang *et al.*, 2015).

Originally, PRP was defined as "the volume of autologous plasma having a platelet concentration above the basal line". It was later defined as a group of soluble and diffusible polypeptide substances able to regulate the growth, differentiation and phenotype of numerous cell types (Vega *et al*., 2000). PRP is an autologous source of many factors and substances, especially platelet-derived growth factor and transforming growth factor-β. These growth factors released after platelet degranulation are those that provide the mechanisms required to produce the necessary biological synthesis for the tissue regeneration process (Grageda, 2004; Grageda *et al*., 2005).

A normal blood clot contains 93% red blood cells, 6% platelets and, in some cases, less than 1% of white blood cells. In contrast, a PRP clot contains 94% of platelets, only 5% of red blood cells and 1% of white blood cells. This change in the ratio of cells that do not stimulate regeneration (red blood cells) vis-à-vis those that do stimulate all phases of regeneration (platelets), explains its ability to reinforce tissue regeneration (Anitua *et al*., 2004; Marx, 2004; Marx & Garg, 2005; Sampson *et al*., 2008). The primarily biostimulation function of PRP is directly linked to the platelet release of growth factors. These factors have tissue regeneration inducing properties. 

In their alpha granules, platelets contain a cocktail of chemical mediators that trigger the onset of the regenerative phase, both through the promotion of angiogenesis and the initiation of cell regeneration by mitotic mediators of mesenchymal cells, which is the "kick off cocktail ". Platelet granules' action is key in the move from the haemostatic to the regenerative phase. Their lysosomes contain many proteolytic enzymes, their dense granules contain prothrombotic factors and their α-granules a high content of pro-regenerative growth factors, among them PDGF and TGF-β. In addition, throughout their formation, platelets have the property of accumulating, by endocytosis, numerous molecules from the medium, among which are lipid mediators, such as sphingosine-1-phosphate (SPP), phosphatidic acid and lysophosphatidate. These substances have an anti-apoptotic effect on endothelial cells, they are involved in the chemotaxis of endothelial cells, and their proliferation and promotion of adhesion bonds form capillary-like structures. Sphingosine appears to be critical to the onset of angiogenesis, since it is the ligand of endothelial differentiation gene (EDG) receptors. Finally, the overexpression of VEGFR2 renders the endothelium sensitive to the production of VEGF (vascular endothelial growth factor) to initiate angiogenesis from endothelial cells (Romagnani *et al*., 2004; Paiva *et al.*, 2011).

Growth factors are obtained by genetic and recombinant engineering procedures; however, they are expensive and repeated doses are required to achieve an optimal therapeutic effect. In contrast, PRP is easy to obtain, it is of low cost and contains a high concentration of growth factors, thus providing an excellent alternative source. Since PRP is an autologous preparation, therefore non-toxic and non-allergenic, it can be used in various medical conditions, as an adjuvant to conventional treatment with mostly satisfactory results. In the case of the endometrial preparation for ART and specifically on the refractory endometrium, not enough studies have been carried out; however, the few reported results demonstrate improvements with its use, in relation to an increase of endometrial thickness, as well as, an increase in implantation rates.

## OBJECTIVE

To improve endometrial quality and implantation rates after the administration of platelet-rich plasma in patients with refractory endometrium undergoing *in vitro* fertilization + embryo transfer treatment in assisted reproduction units (UNAMATER) and (IVF Caracas) in the period from February 2016 to February 2017.

## MATERIAL AND METHODS

### Type of research

Prospective Design.

### Physical environment

This study was carried out in the Maternal Reproductive Assistance Unit (in Spanish UNAMATER) in Merida, Venezuela and at the Venezuelan Institute of Fertility Caracas (in Spanish IVF Caracas) in Caracas D.C, Venezuela.

### Study subjects

The study population consisted of 19 infertile patients undergoing High Complexity ART (*In Vitro* Fertilization), aged between 33 and 45 years with a previous history of refractory endometrium and at least one failed IVF attempt. To these patients, an autologous PRP serum was applied in a quantity of 1 cc by infusion with a catheter into the uterine cavity, on the tenth day of HRT with Estradiol Valerate, and again at 72 hours of the first administration (day 12 of HRT). Endometrial thickness was determined by transvaginal ultrasound.

### Time frame

This study was carried out for one (1) year, from February 2016 to February 2017.

### Selection criteria

#### Inclusion criteria

All patients with failed IVF, either performed in our Units of Human Assisted Reproduction or in External Units, who expressed a desire to undergo a new IVF+ET treatment, in whom preliminarily refractory endometrium was detected. During the period from February 2016 to February 2017.

### Exclusion criteria

- Patients without unsuccessful IVF attempts.
- Patients with previous IVF but without refractory endometrium.

### Resources

#### Human Resources

Clinicians, Embryologists, Anaesthesiologists, Nurses, Cardiologist, Psychologist, among others.

### Institutional Resources

Assisted Human Reproduction Units: Maternal Reproductive Assistance Unit (UNAMATER) in Merida, Venezuela and Venezuelan Institute of Fertility Caracas (IVF Caracas) in Caracas D.C, Venezuela.

### Data collection

The data was collected in a form especially designed for this study.

The patients included in this study consented with their signature and that of a witness; an additional written Informed Consent was used for the application of autologous PRP serum.

### Statistical analysis of the information

After completing collection, the data was transcribed to a database and statistically analysed using the SPSS software version 20.0. The descriptive statistical results are presented in the form of tables of frequency distribution, figures and measurements.

## RESULTS

In the population studied, endometrial thicknesses >7 mm was obtained with the first application of PRP, and in all cases endometrial thicknesses greater than 9 mm became evident after the second application of the autologous preparation. The entire study group qualified for ET in the blastocyst stage.

Of a total of 19 patients with refractory endometrium who underwent IVF+TE during the period between February 2016 and February 2017 and who received two PRP applications as adjuvant in the endometrial preparation, 14 positive pregnancy tests, which corresponded to 73.7%; and 5 presented negative pregnancy tests, which was equivalent to 26.3%. The average age of the patients studied was 39.21±3.22 years (range 33-45 years). When categorizing age values into two age groups, 57.9% (n=11) were grouped around 33-39 years and 42.1% (n=8) aged 40-45 years (Table 1).

**Table 1:** Patient Age

Age	No. of Patients	%	% collected
33	1	5.3	5.3
34	1	5.3	10.5
36	2	10.5	21.1
38	4	21.1	42.1
39	3	15.8	57.9
40	2	10.5	68.4
41	2	10.5	78.9
42	1	5.3	84.2
44	2	10.5	94.7
45	1	5.3	100.0
Total	19	100.0	

With regards to the number of previous IVF attempts, 57.9% (n=11) had had one (1) previous IVF; followed by 31.6% with 2 previous IVF and 10.5% with 3 previous IVF (Table 2). In relation to oocyte origin, 68.4% (n=13) used their own oocyte and 31.6% (n=6) used oocyte donation. (Table 3).

**Table 2:** Previous *in-vitro* fertilization attempts

IVF	No. Patients	%	% collected
1	11	57.9	57.9
2	6	31.6	89.5
3	2	10.5	100.0
Total	19	100.0	

**Table 3:** Type of Oocytes

Oocytes	No. of Patients	%
Own	13	68.4
Donated	6	31.6
Total	19	100.0

As for the type of pregnancy, 26.3% (n=5) presented healthy live births; 26.3% (n=5) had ongoing clinical pregnancies with satisfactory evolution; 10.5% (n=2) had biochemical pregnancies; 5.3 % (n=1) corresponded to anembryonic gestation and 5.3% (n=1) had fetal death at 16 weeks, for which the results of the embryogenic study were expected (Table 4).

**Table 4:** Type of pregnancy

	No. of Patients	%
Ongoing pregnancies	5	26.3
Live births	5	26.3
Biochemical pregnancies	2	10.5
Anembryonic pregnancy	1	5.3
Fetal death (16 weeks)	1	5.3
Total	14	73.7
No pregnancy	5	26.3
Total	19	100.0

When crossing the system of variables regarding oocyte origin and the number of previous IVF, 47.4% (n=9) of the patients with their own oocytes *vs.* 10.5% (n=2) of oocyte receptors had had one previous IVF. 15.8% (n=3) with their own oocytes vs. the same percentage (n=3) of oocyte receptors had had 2 previous IVF and an equal percentage of 5.3% (n=1) for each group presented had had 3 previous IVF (Table 5). When contrasting the type of oocytes with the result of the pregnancy test, 42.1% (n=8) corresponded to a positive test in patients using their own ovules and 31.6% (n=6) had positive tests in oocyte receptors (the total of these had a positive pregnancy test). A 26.3% of the patients using their own oocytes had negative tests (Table 6).

**Table 5:** Previous in-vitro fertilization attempts vs. Type of oocyte

Previous *in-vitro* fertilization attempts	Type of oocyte				Total	
	Own		Donated			
	No.	%	No.	%	No.	%
1	9	47.4	2	10.5	11	57.9
2	3	15.8	3	15.8	6	31.6
3	1	5.3	1	5.3	2	10.5
Total	13	68.4	6	31.6	19	100.0

**Table 6:** Type of Oocytes vs. Pregnancy test

Type of Oocytes	Pregnancy test				Total	
	Positive		Negative			
	No.	%	No.	%	No.	%
Own	8	42.1	5	26.3	13	68.4
Donated	6	31.6	0	0.0	6	31.6
Total	14	73.7	5	26.3	19	100.0

Regarding oocyte origin and its relation to the type of pregnancy, 21.4% (n=3) correspond to ongoing clinical pregnancies in patients using their own oocytes, 14.3% (n=2) for ongoing clinical gestation in oocyte receptors, 14.3% (n=2) of live births with their own oocytes vs. 21.4% (n=3) in oocyte receptors. Same percentage of 7.1% (n=1) for each biochemical gestation group. For anembryonic gestation we had 7.1% (n=1) and 7.1% of fetal death (n=1) was present only in the group of patients using their own oocytes (Table 7).

**Table 7:** Type of oocytes vs. type of pregnancy

Type of pregnancy	Type of oocytes				Total	
	Own		Donated			
	No.	%	No.	%	No.	%
Ongoing pregnancies	3	21.4	2	14.3	5	35.7
Live births	2	14.3	3	21.4	5	35.7
Biochemical pregnancies	1	7.1	1	7.1	2	14.3
Anembryonic pregnancy	1	7.1	0	0.0	1	7.1
Fetal death (16 weeks)	1	7.1	0	0.0	1	7.1
Total	8	57.1	6	42.9	14	100.0

In relation to the number of previous IVF and the result of the pregnancy test a 42.1% (n=8) with a positive test had a history of (1) previous IVF, 15% (n=3) with a negative pregnancy test. A 21.1% (n=2) with positive test presented (2) previous IVF *vs.* 10.5% (n=2) with negative pregnancy test. Finally, 10.5% (n=2) of the patients with positive pregnancy tests presented a history of (3) IVF previous vs. no case in patients with negative pregnancy tests (Table 8).

**Table 8:** Previous *in-vitro* fertilization attempts vs. Pregnancy test

Previous *in-vitro* fertilization attempts	Pregnancy test				Total	
	Positive		Negative			
	No.	%	No.	%	No.	%
1	8	42.1	3	15.8	11	57.9
2	4	21.1	2	10.5	6	31.6
3	2	10.5	0	0.0	2	10.5
Total	14	73.7	5	26.3	19	100.0

Regarding the type of pregnancy related to the number of previous IVF, the highest percentages of 21.4% (n=3) and 14.3% (n=2) of ongoing clinical pregnancies and live births corresponded to patients with one (1) previous IVF, followed by 14.3% (n=2) of live births and ongoing clinical pregnancies in patients with two (2) previous IVF. Biochemical pregnancies with 14.3% (n=2) and anembryonic pregnancy of 7.1% (n=1) in patients with (1) previous IVF. Fetal death at 16 weeks added up to 7.1% (n=1) in patients with three (03) previous IVF. (Table 9).

**Table 9:** Previous *in-vitro* fertilization attempts vs Type of pregnancy

Type of pregnancy	Previous *in-vitro* fertilization attempts						Total	
	1		2		3			
	No.	%	No.	%	No.	%	No.	%
Ongoing pregnancies	3	21.4	2	14.3	0	0.0	5	35.7
Live births	2	14.3	2	14.3	1	7.1	5	35.7
Biochemical pregnancies	2	14.3	0	0.0	0	0.0	2	14.3
Anembryonic pregnancy	1	7.1	0	0.0	0	0.0	1	7.1
Fetal death (16 weeks)	0	0.0	0	0.0	1	7.1	1	7.1
Total	8	57.1	4	28.6	2	14.3	14	100.0

When referring to patient age in relation to the number of oocytes, the highest percentage was 52.6% (n=10) of own oocytes in the age group between 33-39 years old and 15.8% (n=3) of own oocytes in the group between 40 - 45 years. A 5.3% (n=1) oocyte donation in the age group of 33-40 years vs. 26.3% (n=5) oocyte donation in the age group of 40 - 45 years (Table 10).

**Table 10:** Patients age vs. Type of oocytes

Age	Type of oocytes				Total	
	Own		Donated			
	No.	%	No.	%	No.	%
33-39	10	52.6	1	5.3	11	57.9
40-45	3	15.8	5	26.3	8	42.1
Total	13	68.4	6	31.6	19	100.0

When contrasting the age of the patients and the number of previous IVF, 42.1% (n=8) of the patients aged between 33-39 years had previous IVF, 10.5% (n=2) of the same age range group presented 2 previous IVF and 5.3% (n=1) of patients presented 3 previous IVF. Similarly, a 15.8% (n=3) of the patients between 40-45 years had a history of 1 previous IVF, 21.1% (n=4) of this age group presented 2 previous IVF and a 5.3% (n=1) had 3 previous IVF procedures (Table 11).

**Table 11:** Patient age vs Previous *in-vitro* fertilization attempts

IVF	Age				Total	
	33-39		40-45			
	No.	%	No.	%	No.	%
1	8	42.1	3	15.8	11	57.9
2	2	10.5	4	21.1	6	31.6
3	1	5.3	1	5.3	2	10.5
Total	11	57.9	8	42.1	19	100.0

Regarding the age of the patient in relation to the result of the Pregnancy Test, 42.1% (n=8) of the patients between 33-39 years of age had a positive test compared to 15.8% (n=3) in this age group that had negative pregnancy tests. The 31.6% (n=6) of patients between 40 - 45 years of age had a positive test versus 10.5% (n=2) of negative pregnancy tests in the same age group (Table 12).

**Table 12:** Patient age vs. Pregnancy test

Test	Age				Total	
	33-39		40-45			
	No.	%	No.	%	No.	%
Positive	8	42.1	6	31.6	14	73.7
Negative	3	15.8	2	10.5	5	26.3
Total	11	57.9	8	42.1	19	100.0

Finally, when correlating the age of the patients and type of pregnancy, we found that the highest percentages in both age groups corresponded to ongoing clinical pregnancies and live births, with a 14.3% (n=2) and a 21.4% (n=3) in the age group between 33-39 years. 21.4% (n=3) a 14.3% (n=2) respectively, for the age group 40-45 years. Biochemical gestation in the same percentage 7.1% (n=1) for both age groups. A 7.1% (n=1) anembryonic pregnancy and a fetal death of 7.1% (n=1) occurred only in the 33-39 age group (Table 13).

**Table 13:** Patient age vs. Type of pregnancy

Type	Age				Total	
	33-39		40-45			
	No.	%	No.	%	No.	%
Ongoing pregnancies	2	14.3	3	21.4	5	35.7
Live births	3	21.4	2	14.3	5	35.7
Biochemical pregnancies	1	7.1	1	7.1	2	14.3
Anembryonic pregnancy	1	7.1	0	0.0	1	7.1
Fetal death (16 weeks)	1	7.1	0	0.0	1	7.1
Total	8	57.1	6	42.9	14	100.0

## DISCUSSION

The main objective of this study was to improve endometrial quality and implantation rates after the administration of platelet-rich plasma in patients with refractory endometrium undergoing *in vitro* fertilization + embryo transfer treatment. In this sense, the detection performed for one year, showed improvements regarding endometrial thickness in the population studied, after the use of the autologous PRP. Regarding the implantation rates expressed in positive pregnancy tests, 73.7% (n=14) presented very satisfactory results, matching the findings obtained in the few previous serial clinical studies on the matter.

The main advantage of this detection would be the availability of an autologous resource of low cost, which offers obvious benefits regarding the development of a receptive endometrium that favours embryo implantation. Chang *et al.* (2015), in a clinical series of 5 patients with endometrial factor associated with ET cancellation, performed PRP in addition to conventional HRT, achieving in all cases endometrium >7 mm, the total was submitted to ET with subsequent positive pregnancy tests in all patients. These results agree with the results obtained in our study. Garcia-Velasco *et al.* (2016) performed an extensive literature review on the management of refractory endometrium. The review included studies with conventional hormone preparations (estradiol valerate), as well as various drugs including acetylsalicylic acid (aspirin), sildenafil, vitamin E, GnRH agonists, HCG, l-arginine, pentoxifylline among others; as well as autologous preparations as growth factors, mainly G-CSF, stem cells, PRP and bone marrow. Finally, they concluded that despite the vast array of resources available today, it is still not easy to provide a pragmatic evidence-based approach that guides the clinician on how to improve refractory endometrium. In this sense, and in relation to the results of this study, it is considered important to complement them with the formal design of test protocols of the test versus test type, as well as, comparative studies to establish the efficacy between different drugs and preparations, besides the inclusion of several years of data collection.

## CONCLUSIONS AND RECOMMENDATIONS

PRP administration as an adjuvant in the endometrial preparation of patients with refractory endometrium, demonstrated a good sensitivity for increasing endometrial thickness, as well as, to obtain positive pregnancy tests, clinical pregnancies and live births. The use of the PRP preparation and its biostimulation effects on the endometrium microvascular endothelium seems to offer benefits to refractory endometrium, providing an increase in endometrial receptivity and a consequent increase in implantation rates. Being an autologous resource, therefore harmless to the patient, easy to obtain and of very low cost, it is recommended to be included in the different protocols for endometrial preparation in assisted reproduction techniques, even in those where the natural cycle is preferred, that is, without the requirement of hormone replacement therapy.

Further studies are recommended in terms of the number of population under study and the time frame evaluated, as well as the approach of comparative studies between drugs and autologous preparations, to envisage effective therapeutic alternatives for refractory endometrium. Despite having the latest resources, uterine transplantation, something unimaginable a while ago, arises today in response to fertility preservation as far as uterine factor is concerned. It is necessary to establish preliminary protocols with the use of a wide range of therapeutic alternatives, within which the autologous preparations are becoming increasingly important today based on the positive results obtained.
